# Digenic Analysis Finds Highly Interactive Genetic Variants Underlying Polygenic Traits

**DOI:** 10.18103/mra.v11i10.4604

**Published:** 2023-10-30

**Authors:** Gao Wang, Jurg Ott

**Affiliations:** Columbia University, New York; Rockefeller University, New York

## Abstract

We briefly review our recently published approach to mining digenic genotype patterns, which consist of two genotypes each originating in a different DNA variant. We do this for a genetic case-control study by evaluating all possible pairs of genotypes, distributing the workload over numerous CPUs (threads) in a high-performance computing environment and apply our methods to two known datasets, age-related macular degeneration (AMD) and Parkinson Disease (PD). Based on a list of (e.g., 100,000) genotype pairs with largest genotype pair frequency differences between cases and controls, we determine the number $N_{u}$ of unique variants occurring in this list. For each unique variant, we find the number of genotype pairs it participates in, which identifies a set of variants “connected” with the given unique variant. Among the total of variants “connected” with all unique variants, only a subset of variants is unique. The ratio of all connected variants divided by that subset of variants is a measure for the overall density or connectedness of variants interacting with each other. We find that variants for the AMD data are much more interconnected than those for PD, at least based on the 100,000 genotype pairs with largest chi-square we investigated. Further, for each of the $N_{u}$ unique variants, we use the number of variants connected with it as a test statistic, weighted by the inverse of the rank at which the unique variant first occurred in the original list of genotype patterns. This weighing scheme ties the number of connections to the genetics of the trait and allows us to obtain, for each of the $N_{u}$ unique variants, an empirical significance level by permuting ranks. We find 12 and 8 significant, highly connected variants for AMD and PD, respectively, some of which have previously been identified by other machine learning methods, thus providing credence to our approach. Among the 100,000 genotype pairs investigated for each of AMD and PD, significant variants showed connections with up to 7,093 and 3,777 other variants, respectively. Our approach has been implemented in a freely available piece of software, the *Digenic Network Test*. Thus, our statistical genetics method can provide important information on the genetic architecture of polygenic traits.

## 2.0 Introduction

Genetic mapping of genes responsible for observed traits arguably has its origins in the early 1900s in the “fly room” at Columbia University^1^. Alfred Sturtevant, then an undergraduate, experimented with offspring of planned crosses of the fruit fly and developed on the X chromosome the first genetic map, consisting of the order and approximate linear spacing of six genes that is still valid today^2^. In humans, genetic linkage analysis based on family pedigrees^3^ has for many years provided localizations for disease genes, and so have the currently favored genome-wide association studies (GWASs)^4^. However, many of these approaches analyze correlation between disease phenotype and one DNA variant (SNP, single-nucleotide polymorphism) at a time, yet genes are unlikely to act independently but operate in concert with many other genes and environmental conditions. Here we introduce a new method for genetic trait mapping based on interactions among SNPs, which allows us to obtain information on the genetic architecture on two levels, (1) in a rather general way and (2) in more detail for each of the statistically significant SNPs. These significant SNPs can point to genes or pathways underlying the disease process and are potentially useful as drug targets in the treatment of disease.

As early as 30 years ago, two-locus models of disease inheritance have been considered in human genetics^5^ and implemented in computer programs^6^ but they were cumbersome and inefficient because of the presence of various unknown parameters in these models. Considerable progress was achieved when machine-learning methods were implemented in the analysis of human case-control data, and numerous different approaches to multi-variant analysis were published^7–10^ although most of these approaches are applicable only to rather small datasets. Recently, efficient software has been developed that makes use of multiple processors (CPUs, threads) in current Windows and Linux workstations^11^. Based on long lists of genotype pairs resulting from the analysis of case-control data under a digenic model, we now present a novel approach to uncover large numbers of interactions between a given significant variant and many others, where individual variants may not show much effect. This approach is likely to find highly significant relationships between variants while other approaches may fail.

## 3.0 Methods

Consider a genetic case-control dataset in *plink*^12^ format, that is, a *map* file holding information like chromosome number and base-pair position for each variant (SNP), and a *ped* file listing genotypes at each variant for a given individual. For illustration purposes, we will be working with datasets on two traits, Parkinson Disease (PD)^13^ with 541 individuals (270 cases, 271 controls), each genotyped at 379,502 variants (downloaded in 2006 from the NINDS Coriell Institute, see Acknowledgments), and age-related macular degeneration data (AMD)^14^ with 146 individuals (96 cases, 50 controls), each genotyped at 103,611 variants (AMD data available at https://www.jurgott.org/linkage/GPMdata.zip).

Initially, a conventional GWAS is carried out with the *Cochran-Armitage trend test*^15^ as implemented in *plink*, that is, we test for each SNP whether the trend in genotype frequencies across genotypes is different in cases and controls. Any variants turning up significant (permutation test) will be removed from further analysis as we are mainly interested in interactions among variants rather than their individual (main) effects^7^.

Consider two SNPs, each with three genotypes, so that there are nine genotype pairs for the two SNPs. We want to evaluate all possible genotype pairs in a given dataset and list, for each genotype pair (called a *genotype pattern*), its frequency in cases and controls. This is accomplished by the *Gpairs* program^11^, which will, for each genotype pair, create a 2 × 2 table of individuals (see Table 1 in Ott & Park^16^), with rows representing cases and controls, and columns referring to presence and absence of the given genotype pattern in an individual. We generally disregard genotype patterns occurring in fewer than *s* individuals (so-called *support* for the pattern), and the two SNPs furnishing genotype patterns are required to reside on different chromosomes so as to avoid any potential interference with linkage disequilibrium^11^. Each 2 × 2 table will be analyzed by a 2-sided version of the Irwin-Fisher exact test^17^, that is, we are interested in whether a pattern occurs more often or less often in cases than controls. The Fisher test furnishes for each table a *p*-value (transformed to chi-square for easy interpretation), whose associated corrected empirical significance level may be obtained with the Bonferroni correction, $p_{Bon}=Np$, where N is the number of tests performed. This procedure generally furnishes long lists of genotype pairs. Often, however, few if any genotype pairs show statistically significant frequency differences between cases and controls, $p_{\text{Bon}}\leq 0.05$. This is in a nutshell a description of our current approach to finding pairs of genotypes (digenic patterns) and their frequencies in cases and controls^11^.

Testing for frequency differences of digenic patterns between cases and controls tends to have low power, partly due to the current requirement of applying Bonferroni correction for multiple testing (dramatic increases in computing power may change this situation in the future). Thus, we developed the *digenic network test* (DNT), which implements an exploration of digenic data from a different angle. Our novel approach to interpreting disease-predisposing genotype patterns starts with a possibly very long list of genotype patterns, with each pattern being characterized by chi-square from the Fisher test mentioned above. Assume that such a list is ordered so that the genotype pair (and corresponding variant pair) with largest chi-square is ranked 1, and we want to retain, for example, only the best $N_{\text{pairs}}=100,000$ genotype pairs, that is, the pairs ranked 1 through $N_{\text{pairs}}$. Focusing on variants rather than their genotypes, we observe that a given variant occurs in multiple lines of the list. In other words, a given variant may be connected with various other variants. There will be $2\;\times N_{\text{pairs}}$ variants in the list, but many variants occur multiple times, and we are interested in variants being connected with large numbers of other variants.

Thus, we prepare a list of unique variants occurring in the $N_{\text{pairs}}$ variant pairs. Some of the resulting $N_{u}$ unique variants are connected with large numbers of other variants, while many unique variants show only one connection. For each unique variant, we record its number of connections, $c_{i}=1,\ldots ,N_{u}$, and the rank, $r_{i}$, at which the variant first occurred in the ordered list. The total number of variants connected with the $N_{u}$ variants is $S_{1}=\Sigma_{i}c_{i},i=1\ldots N_{u}$, but some of these $S_{1}$ variants may be connected with more than one of the $N_{u}$ variants. We therefore determine $S_{2}$, the number of unique variants among the $S_{1}$ variants, where $S_{2}\leq S_{1}$. If the $S_{1}$ variants are all different from each other, then each of the $N_{u}$ variants points to a different set of connected variants and $S_{2}=S_{1}$. On the other hand, if many of the $S_{1}$ variants are being pointed to by multiple of the $N_{u}$ variants, then $S_{2}<<S_{1}$ and the set of $N_{u}$ plus $S_{2}$ variants represents a dense collection of interacting variants. Thus, the ratio, $=S_{1}/S_{2},$ R≥1, is a measure for the overall density or connectedness of the interacting variants.

To evaluate whether large numbers of connections are related to the genetics of the trait, that is, whether they are enriched in top-ranked variants, we define a test statistic, $T_{i}=c_{i}/r_{i},\;i=1,\ldots ,N_{u}$, so that the number of connections is weighted by the inverse of the rank of each unique variant. Statistical significance of each of the $N_{u}$ unique SNPs is obtained by permutation analysis in that we permute all ranks $N_{\text{perm}}=100,000$ times and each time record the largest $T_{i}$ value, $T_{i,\text{max}},\;i=1,\ldots ,N_{\text{perm}}$. The proportion of $T_{i,max}$ values at least as large as an observed $T_{i}$ represents its associated empirical significance level, $p_{i},\;i=1,\ldots ,N_{u}$. Software (program DNT) to carry out these calculations is freely available at: https://www.jurgott.org/linkage/DNT.html. As will be seen below, in each of our two sample datasets, some of the $N_{u}$ unique variants are highly significant and are thus called lead SNPs or lead variants^4^. This approach allows us to find connections between variants significantly related to genetic effects (chi-square) although genotype pattern frequencies may not be significantly different between cases and controls.

## 4.0 Results

We demonstrate our methods for the two published datasets, AMD and PD, mentioned in section 3.0. For PD, we use all variants while we disregard two variants (rs380390 and rs1329428) in the AMD dataset because they are significant in our single-variant trend test (in the original publication^14^, rs380390 and rs10272438 were significant). Of course, the AMD dataset is smaller than PD, both in terms of variants and numbers of individuals. For each dataset, we applied the *Gpairs* program^11^ to generate pairs of genotypes (patterns). To be considered for further analysis, a pattern had to occur in at least 20 individuals, and the two genotypes had to come from variants on two different chromosomes

For the AMD and PD datasets, Tables 1 and 2 respectively show the relatively small number of lead variants connected with large numbers of other variants in the 100,000 genotype patterns with largest chi-square values. Statistical significance in these tables refers to our test statistic, T, but the variants are listed in chromosomal order. Clearly, these data demonstrate large networks of variants associated with disease. For AMD, a total of $S_{1}=30,935$ variants are connected with 12 lead variants extracted by our procedure from the best 100,000 genotype patterns. As there is some overlap among connections between the latter variants and the $S_{1}$ variants, the number of unique variants among the $S_{1}$ variants is only $S_{2}=19,128$ (obtained in a spreadsheet). Thus, our measure for dispersion among connected variants is $R=S_{1}/S_{2}=1.62$ for the AMD data. On the other hand, for the PD data, $S_{2}=9,340$ and R=1.12 – much smaller than the R value obtained for AMD. Thus, within the best 100,000 genotype pairs, variants in the AMD dataset are more connected among themselves than in the PD data.

If the full AMD dataset is analyzed, including variants rs380390 and rs1329428, then we obtain even larger numbers of connections to other variants, that is, 20,814 connections for rs380390, and 17,365 connections for rs1329428.

Also shown in Tables 1 and 2 is the rank (“rank GWAS”) of each lead SNP in the trend test performed by *plink*. Clearly, some of the lead SNPs have very small main effects, that is, small frequency differences in genotypes or alleles between cases and controls, as indicated by ranks exceeding 10, for example.

**Figure 1:**
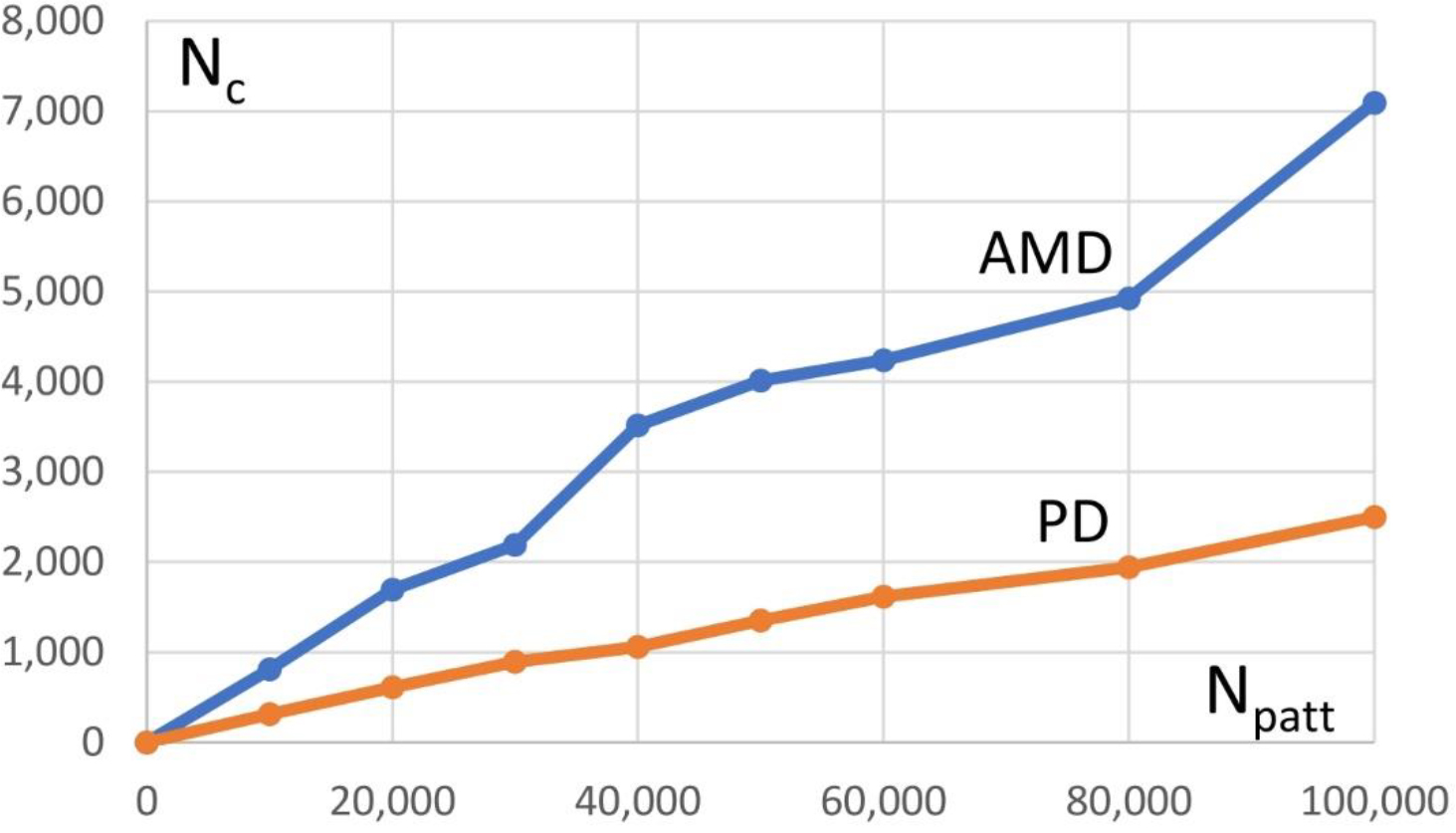
Number $N_{c}$ of variants connected with the most significant variant in each of the AMD and PD datasets within the most significant $N_{patt}$ genotype patterns.

The difference in connectedness between the two datasets may also be seen when we consider only the most significant variant in each of tables 1 and 2 and follow its increase in the number $N_{c}$ of connected variants with an increasing number $N_{patt}$ of most significant genotype patterns. As Figure 1 shows, AMD data exhibit a much stronger increase in connectedness than PD data for each of their most significant variants. It is also clear that these curves are far from reaching a plateau yet, but we have been able to demonstrate genetically that at least among the most significant genotype patterns, there are many more significant connections among variants in AMD than PD. It may well happen that this situation is reversed when even larger numbers of genotype patterns are analyzed, but we have not looked into that situation.

## 5.0 Discussion

In one of our previous publications^16^, we discussed the rationale for working with patterns rather than single variants and also outlined applications to individual identification. Here, based on large numbers of genotype pairs (and associated variant pairs), we developed an approach to building significant networks of variants that are related to the discrimination between cases and controls.

The main difference between GWAS approaches and our method is that GWASs assess individual (main) effects of each SNP while we work directly with interactions between any two genotypes and, thus, SNPs. One of the currently favored genetic constructs, polygenic risk scores (PRSs), combine information over many or all SNPs in a GWAS^4^. For example, in psychiatric genetics, polygenic scores can predict behavioral and medical outcomes^18^ even though these scores capture “only” main effects of the SNPs they comprise. At times, however, PRSs (that is, genetic variants) add little to clinical and environmental risk indices^19^. On the other hand, a multi-PGS framework has been proposed that combines hundreds of PRSs obtained from publicly available GWASs and can result in increased phenotype prediction^20^.

Our test statistic for each of the $N_{u}$ unique variants in the best 100,000 genotype pairs consists of two parts, the number of connections to other variants and a weight, which should reflect the genetic “loading” associated with the number of connections. Here, we have chosen as a weight the inverse of the rank, at which a unique variant first occurs in the long list of genotype pairs. An alternative weight would be the chi-square value obtained from the Fisher test for each unique variant. There may be other conceivable weighing schemes, but we have not pursued this further as the current setup has furnished remarkable results.

### 5.1 AGE-RELATED MACULAR DEGENERATION

The AMD dataset has been used widely to illustrate new statistical procedures, notably techniques involving multiple variants. Early-on, methods have been developed to find novel variants correlated with known risk variants. For example, rs10511467 has been identified in this manner based on a specific search algorithm^21^. After removal of five variants with strong main effects, three of our variants (rs1363688, rs7104698, and rs1394608) were found with a search algorithm although some results are not statistically significant^22^. More recently, rs1363688 was found with a method related to genetic algorithms although the search included variants in known risk loci^23^.

As pointed out above, our approach avoids using known risk variants and works exclusively with genotype patterns found in an exhaustive search. The fact that our highly significant results confirm previous findings should provide additional confidence in our method so that variants listed in Table 1, but not found in the literature, deserve careful attention, but they are not followed up here.

### 5.2 PARKINSON DISEASE

A recent review of PD genetics lists various known risk genes but the three genes mentioned in Table 2 are not in that list^24^. However, emerging multi-omics resources and analyses related to PD could provide support for novel genes identified through our Digenic Network Test. Specifically, a recent study reported a strong mRNA expression difference for *IL2RB* between PD cases and controls in females (*p* < 0.0001) but not in males (*p* = 0.8013)^25^. This result validates our finding for variant rs229492 in Table 2.

Several other variants in Table 2 have previously been reported. Variants rs4862792 and rs1480597 were already mentioned in the publication providing the dataset analyzed here^13^, and variants rs243023 and rs1480597 were detected by a specific tree classifier^26^. In a recent GWAS meta-analysis for PD, two of our eight significant variants (rs4862792 and rs1480597) were confirmed^27^. Variant rs4862792 on chromosome 4 is located within 24 KB of the LOC339975 gene and was reported as a nearly significant risk variant for major depressive disorder (MDD) in a large case-control study^28^, indicating a relationship between MDD and PD, which has recently been reviewed^29^.

## 6.0 Conclusions

The conventional GWAS approach has proven to be a powerful tool in identifying genes associated with disorders where a single gene variation plays a major role. However, many conditions are shaped by several genes, and sometimes these genes might not show strong effects by themselves. In our study of two diseases, AMD and PD, which have been extensively researched using GWAS, we developed a novel statistical method which aggregates patterns of gene-pairs related to disease into networks, suggesting potential genetic interactions that influence these diseases. The results presented in this paper highlight the initial success of the new approach.

## Figures and Tables

**Table 1.** Twelve variants significantly connected with other variants based on 100,000 genotype patterns in the AMD dataset.

r	lead SNP	c	T = c/r	p	chr	bp	rank GWAS	Gene	Function
14	rs551591	1,495	106.8	0.0171	1	52,836,204	39,414	—	—
1	rs475553	162	162.0	0.0104	3	165,658,332	292	—	—
22	rs7698633	1,243	56.5	0.0381	4	84,060,998	859	LINC02994	iva
3	rs4920799	158	52.7	0.0415	5	85,310,710	4	—	—
16	rs1394608	5,619	351.2	0.0038	5	156,356,284	53	SGCD	iva
5	**rs1363688**	7,093	1,418.6	0.0006	5	175,182,728	3	—	—
8	rs10486157	500	62.5	0.0337	7	7,311,052	82	LOC107986764	iva
4	rs10488343	302	75.5	0.0267	7	131,889,562	28,085	—	—
15	rs10511467	6,670	444.7	0.0026	9	7,373,051	16	—	—
2	rs7104698	2,748	1,374.0	0.0007	11	36,852,015	8	LOC107984326	gdt, iva
11	rs6104678	3,530	320.9	0.0043	20	10,953,559	197	—	—
20	rs200642	1,415	70.8	0.0286	20	53,326,493	23	TSHZ2	iva
	Sum, *S*_1_ =	30,935							

Notes: *r* = rank, ID = variant identifier, *c* = number of connected variants, *T* = our test statistic, *p* = empirical significance level based on 100,000 permutations of ranks, *chr* = chromosome number, *bp* = basepair position (GRCh38), *Gene* = gene containing the given variant, *iva* = intron variant, *gdt* = genic downstream transcript variant. The most significant variant is shown with ID in bold; it is located within 21.5 MB of the CFH gene.

**Table 2.** Eight variants significantly connected with other variants based on 100,000 genotype patterns in the PD dataset.

r	lead SNP	c	T = c/r	p	chr	bp	rank GWAS	Gene	Function
4	**rs243023**	2,499	624.8	0.0003	2	60,356,592	37	—	—
16	rs4862792	1,778	111.1	0.0057	4	187,280,196	21	—	—
12	rs6918975	3,777	314.8	0.0011	6	21,817,734	1,247	CASC15	iva
7	rs7026302	408	58.3	0.0146	9	130,953,088	176	—	—
43	rs1480597	1,161	27.0	0.0433	10	44,665,661	5	—	—
1	rs7299117	49	49.0	0.0190	12	127,217,415	13,889	—	—
17	rs11620883	426	25.1	0.0467	14	69,255,063	44	GALNT16-AS1	gut, iva
5	rs229492	378	75.6	0.0100	22	37,164,552	50	IL2RB	gut, iva
	Sum, *S*_1_ =	10,476							

Notes: see Table 1, *gut* = genic upstream transcript variant.
